# *Camellia sinensis* in Dentistry: Technological Prospection and Scientific Evidence

**DOI:** 10.1155/2021/9966738

**Published:** 2021-08-30

**Authors:** Lídia Audrey Rocha Valadas, Rosueti Diógenes de Oliveira Filho, Edilson Martins Rodrigues Neto, Mary Anne Medeiros Bandeira, Marta Maria de França Fonteles, Vanara Florêncio Passos, Ana Cristina de Mello Fiallos, Mara Assef Leitao Lotif, Nara Juliana Custodio de Sena, Thereza Cristina Farias Botelho Dantas, Igor Lima Soares, Patricia Leal Dantas Lobo, Aldo Fabian Squassi

**Affiliations:** ^1^Department of Preventive and Community Dentistry, University of Buenos Aires, Buenos Aires, Argentina; ^2^Pharmacy, Dentistry and Nursing College, Federal University of Ceara, Fortaleza, Brazil; ^3^Nova Esperança Nursing College (FACENE), Mossoró, Brazil; ^4^Paulo Picanço College of Dentistry, Fortaleza, Brazil; ^5^Unichristus, Fortaleza, Brazil

## Abstract

**Purpose:**

This study aimed to evaluate reports of patents for oral care formulations, based on *Camellia sinensis* (*C. sinensis*), deposited and granted in intellectual property banks.

**Methods:**

A survey was conducted through collection, treatment, and analysis of extracted information from patent reports selected. The documentary research was conducted in January 2021 on formulations with *C. sinensis* for dental applications, including since the first patent deposits until the current time. The risk of bias of clinical trials with these formulations was analyzed to verify the scientific evidence. The data extracted represent the distribution of the number of patents by banks, annual evolution of patent deposits, applicant of patents by country, distribution of patents according to International Patent Classification codes, and the types of patented products.

**Results:**

Data and information from 20 selected patents were extracted. The United States Patent and Trademark Office (USPTO) and World Intellectual Property Organization (WIPO) were the banks with the largest number of patents for products/formulations with *C. sinensis* for oral care applications with 7 (35%) and 6 (30%) patent registrations, respectively. Other banks did not provide patents related to the search. Patents of compositions were the largest with 14 filings, and the remainder of formulations are represented specially by mouthwashes and toothpastes. As for clinical application, 18 patents were filed as products with antimicrobial and antibiofilm action, while 2 patents are directed to the treatment of xerostomia. In general, the aspects of the studies of clinical efficacy pointed to a low risk of bias.

**Conclusion:**

The study pointed out a small number of products protected by patents for *Camellia sinensis* for oral care indication, highlighting mainly mouthwash compositions and formulations. In the methodological parameters of clinical trials carried out with the formulations, the majority pointed out a low risk of bias.

## 1. Introduction

The practice of using medicinal plants with pharmacological activities for the treatment of diseases is ancient, including for conditions related to oral health. The use of natural products as medicinal therapy existed long before the pharmaceutical industry and after the emergence of these products has been incorporated into the development of formulations to the present day [1–3].

Herbs and medicinal plants can be used in different ways, including whole herb, leaves, roots, essential oils, and prepared as teas, syrups, creams, ointments, and even capsules or pills that contain a powdered form of the plant [4]. Tea constitutes an infusion prepared from dry leaves and is the second most consumed drink in the world, its consumption being surpassed only by water, in addition to having great cultural and economic relevance in several countries. Among the most varied types of teas, *Camellia sinensis* (*C. sinensis*) teas stand out as one of the most popular and used worldwide [5–7].

*C. sinensis* is a plant rich in polysaccharides, caffeine, polyphenols, amino acids, and antioxidants, in addition to micro and macronutrients beneficial to human health [8]. It has different important pharmacological properties such as antimicrobial, antioxidant, antidiabetic, and anti-inflammatory activities, in addition to its consumption presenting several documented benefits related to oral health, especially regarding caries and periodontal diseases [3, 6].

This plant is a species belonging to the Theaceae family and has small perennial shrubs, widely used to produce teas, especially green and black [9]. These teas have several pharmacological properties, with their production being carried out mainly in regions of tropical and subtropical climate, with abundant and regular rainfall [9, 10]. According to its fermentation and maturation process, *C. sinensis* tea can be classified as green, white, yellow, red, and black, where important differences can be verified according to its cultivation and leaf processing [6, 11].

Green tea from *C. sinensis* has a strong antioxidant power through its polyphenolic chemical constituents, beneficial in several clinical conditions such as dental caries, gingivitis, periodontitis, and halitosis, in addition to neuroprotection in the oral cavity [1]. Since it has a high concentration of fluoride in its nutrient composition, the consumption of this tea is extremely beneficial in repairing dental tissue in an acidic environment and improving resistance to demineralization [12, 13]. Furthermore, it has been shown that this tea has other properties, such as antiviral action against influenza viruses, herpes viruses, and antifungal action against *Candida albicans*, *Trichophyton mentagrophytes*, and *Trichophyton rubrum* [14].

Black tea is a product of *C. sinensis* treated with an oxidation process that requires longer steps and contains a greater amount of caffeine when compared to other teas from this plant. Its continued use can reduce blood pressure, the risks of type 2 diabetes, and improve the lipid profile. Its polyphenolic constituents promote health benefits, mainly in obesity, diabetes, cancer, atherosclerosis, inflammatory diseases, and osteoporosis [15–18].

In recent years, the search for new substances and formulations with pharmacological potential and biocompatibility has increased, which is revealed by the growing number of studies on the use of natural products. Often these studies seek to generate new technologies for society with a natural raw material traditionally used, giving rise to the development of innovations and patents [19, 20].

The elaboration of an invention patent or utility model is an extremely relevant indicator to mainly evaluate the level of development and technological innovation in industries and research institutions. Therefore, natural products are sources of biomolecules or therapeutic complexes that can be used for technological innovation and maintain competition in the market in several areas, including products for dental applications [21, 22]. Given the economic importance and medicinal products of *C. sinensis*, as well as its several benefits to systemic and mainly oral health, this study aimed to evaluate reports of patents deposited and granted on dental formulations based on *C. sinensis* in intellectual property banks.

## 2. Materials and Methods

### 2.1. Elaboration of the Technological Prospective Study

The survey was conducted through collection and analysis of extracted information from patent reports selected. The documentary research was conducted in January 2021 about formulations with *C. sinensis* for dental applications, including since the first patent deposit in 2004 until all the year of 2020. The searches were direct with access to reports of patents deposited and granted in the following intellectual property banks of worldwide references:

1. Canadian Intellectual Property Office (CIPO)—Canada
2. China National Intellectual Property Administration (CNIPA)—China
3. Espacenet—European Patent Office (EPO)—Europe
4. German Patent and Trademark Office (DPMA)—Germany
5. Intellectual Property India—India
6. Japanese Patent Office (JPO)—Japan
7. National Institute of Intellectual Property (INPI)—Brazil
8. Swiss Federal Institute of Patent Office (IGE-IPI)—Switzerland
9. United States Patent and Trademark Office (USPTO)—United States
10. World Intellectual Property Organization (WIPO)—Europe

### 2.2. Search Strategy and Data Extraction

For the preparation of the study, we conducted a mapping of patent applications using the keyword “*Camellia sinensis*” in the search field. All patent documents that included this term were initially considered in the search with the exploratory reading of titles and summaries, as a criterion for inclusion of the patents found. Then, only the active reports related to dentistry were selected; expired, abandoned, or denied patents were not included. Relevant information that describes the invention in the patent reports was selected and organized in graphics in GraphPad Prism 6 program to analyze descriptive statistics.

The data extracted represent the distribution of the number of patents by banks, annual evolution of patent deposits, applicant of patents by country, distribution of patents according to International Patent Classification (IPC) codes, and the types of patented products.

### 2.3. Scientific Evidence

According to the patents selected at the end of the search, the described inventions and their purposes with dental applications were evaluated and were searched clinical trials related to each selected patent in PubMed.

### 2.4. Risk of Bias Assessment

Trials were assessed using Cochrane's tool for assessing the risk of bias in randomized trials [23]. The tool includes the following domains: random sequence generation, allocation concealment, blinding of participants and personnel, blinding of outcome assessment, incomplete outcome data, selective reporting, and other sources of bias. We rated each domain as low risk, unclear risk, or high risk of bias.

We classified the overall risk of bias as low if all domains were at low risk of bias, as high if at least one domain was at high risk of bias, or as unclear if at least one domain was at unclear risk of bias, and no domain was at high risk. This rule is specified by the Cochrane tool for assessing the risk of bias in randomized controlled trials because any source of bias in a trial is problematic, and there is a paucity of empirical research to prioritize one domain over the other.

## 3. Results

The initial search resulted in 5126 patents found with the term “*Camellia sinensis*” in the intellectual property banks selected, followed by the stage title and abstract read targeting dental applications from the first patent deposited until the last one that had a total of 28 patent registrations. Then, the repeated records (8) were deleted. At the end of the search, data and information from 20 selected patents were extracted (Table 1).

According to the searches, the United States Patent and Trademark Office (USPTO) and World Intellectual Property Organization (WIPO) were the banks with the largest number of filing patents for products/formulations with *C. sinensis* for dental applications with 7 (35%) and 6 (30%) patent registrations, respectively. The German Patent and Trade Mark Office (DPMA), Japanese Patent Office (JPO), and Swiss Federal Institute of Intellectual Property (IGE-IPI) did not provide patents related to the search.

**Figure 1:**
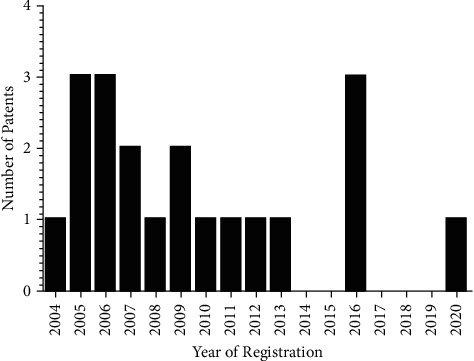
Annual evolution number of patent filings per year with the term *Camellia sinensis* in formulations for oral care applications in the intellectual property banks accessed, 2021.

Figure 1 shows the annual evolution of patent filings according to the number of patent registrations. There is an increase in the number of deposits, with a highlight from 2005 to 2006 with a total of 6 patents, also in 2016 with 3 registrations, and no deposits were found between 2017 and 2019. It is noteworthy that as expected in the last 18 months, the number of patents submitted found in the search is less than reality since many deposits are still in the period of confidentiality.

**Figure 2:**
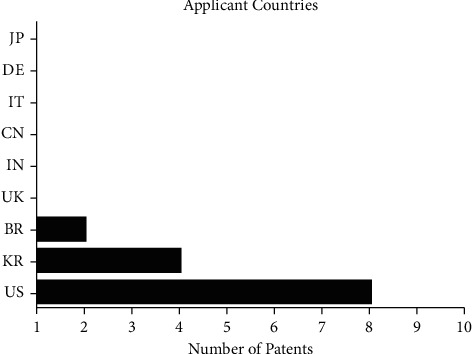
Applicant of patents by country with term “*Camellia sinensis*” in formulations for oral care applications in the intellectual property banks accessed, 2021.

Figure 2 shows the origin of the patent applicants with a wide variety of countries, highlighting the United States as the largest applicant with 8 (40%), followed by South Korea with 4 (20%) and Brazil with 2 (10%) patent filings. Other countries, such as the United Kingdom, Italy, Germany, India, China, and Japan had only one patent registration, representing 5% each.

There were different types of patent applicants according to the findings. We observed that half of the patents filed (10) were inventions developed and registered by companies and industries. Also, the Colgate–Palmolive Company (US) showed most patents with three filings and the others with only one registration, among them the Nippon Zettoc Company, Ltd. (JP), Indena SpA Company (IT), and Amorepacific Corporation (KR). As well as, the applicants were represented by universities and research institutes with seven filings, among them Kingston University (UK), São Paulo University (BR), and Georgia Health Sciences University Research Institute (US). The other patents (3) had their applicants represented by people (inventor) and researchers.

**Figure 3:**
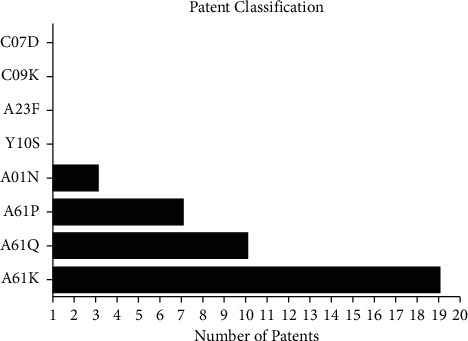
Distribution of patents deposited with term “*Camellia sinensis*” for dental applications by International Patent Classification Codes (IPC). A61K: prescriptions for medical, dental, or hygienic purposes; A61Q: cosmetic products or formulations for personal hygiene; A61P: specific therapeutic activity of chemical compounds or medicinal presets; A01N: preservation of organisms of humans or animals or plants or their parts; A23F: coffee, tea, their substitutes, manufacture, preparation, or infusion thereof; C09K: materials for applications not otherwise provided for; applications of materials not otherwise provided for; C07D: heterocyclic compounds; Y10S: technical subjects covered by former USPC cross-reference art collections (XRACs) and digests.

In Figure 3, there is the distribution of classification of patented products indicating that the patent filings are more focused on human need section (A), and the classification A61K presented the largest number of patents (19), a category which includes patents for the preparations for medical, dental, or hygienic purposes, followed by A61Q with cosmetic products or formulations for personal hygiene (10), A61P with specific therapeutic activity of chemical compounds or medicinal preparations (7), and A01N with preservation of organisms of humans or animals or plants or their parts (3). As we can observe, some reports have more than one classification.

**Figure 4:**
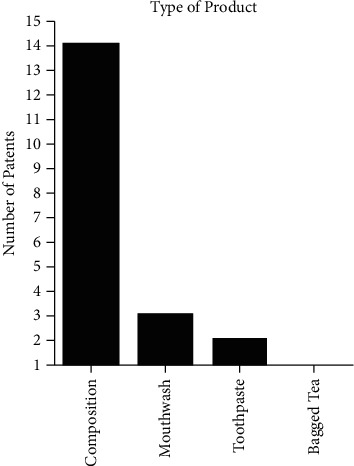
Types of dental formulations deposited with the term “*Camellia sinensis*.” Applicant of patents by country with term “*Camellia sinensis*” in formulations for dental applications in the intellectual property banks accessed, 2021.

Figure 4 shows that the types of patented dental products with the term “*Camelia sinensis*” were the largest in composition form with 14 filings, and the remainder of the formulations is represented in mouthwash (3), toothpaste (2), and bagged tea (1). As for clinical application, 18 patents were filed as products with antimicrobial and antibiofilm action, while two patents are directed to the treatment of xerostomia.

**Figure 5:**
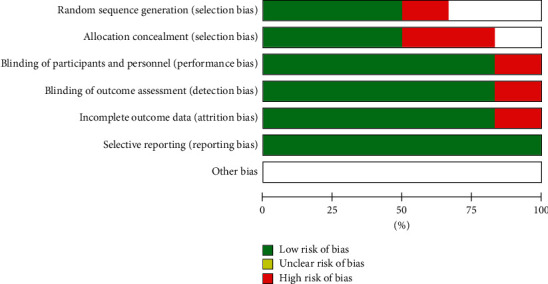
Review authors' judgements about each risk of bias item presented as percentages across all included clinical studies.

Figure 5 shows the level of evidence of clinical trials studies about the efficacy of these formulations, found in the PubMed database. For the risk of bias, factors are random sequence generation, allocation concealment, blinding of participants and personnel, blinding of outcome assessment, incomplete outcome data, selective reporting, and other bias. In general, the risk of bias of the studies was low, reinforcing that the formulations were effective.

## 4. Discussion

In the last decades, the scientific community and industries have shown a growing interest regarding the technological innovation for new products in dentistry, mainly based on the development of patents as intellectual property, which is a response to market demands [20]. Many of these inventions have been developed using natural products, such as *C. sinensis* extract, with different potential benefits for human oral health. Formulations containing *C. sinensis* in its composition look for advantages for treatment of oral biofilm, helps promote the enamel and dentin remineralization by the fluoride component, and bad breath treatment [3, 6, 7, 12, 13, 15, 18].

Two reviews highlight and discuss patents that used *C. sinensis* and its derivatives for different applications according to the available literature. The first review highlighted that several patents were developed to improve tea processing methods for different purposes, including promoting changes in the composition of tea products, improving their sensory properties and stability, and increasing production yield [15]. The second reported invention patents using trihydroxybenzoate derivatives present in the tea composition of *C. sinensis* with antiviral, antifungal, and antibacterial properties related to different diseases, not only to oral health [14]. However, our study highlights recent patents that employed *C. sinensis* in formulations focused exclusively on dental applications, being the first review of patents for oral care on this topic.

The present study observed that the majority of patent deposits with *C. sinensis* belonged to the United States, both as an applicant country and as an intellectual property bank. Therefore, following the ranking of the WIPO statistical base, that when considering the technology areas, the United States is in first place in the medical field with patent deposits, including value-added dental equipment in the economic sector [24]. Among the types of applicants, the Colgate–Palmolive company was already expected to hold of a greater number of deposits, although the amount is not yet significant. Today, it represents a pioneering brand in the production of toothpaste, founded in New York (US) with more than 200 years of existence in the global market and later started to add the soap brand Palmolive.

The first patent registration (PI0519427-0) [25] entitled “Oral composition and methods for promoting the oral health of an animal patient, and for reducing the extension of discoloration in a toothpaste” was deposited in 2004 at the intellectual property bank Espacenet by the Colgate–Palmolive Company. The invention describes low water content toothpaste containing a variety of plant extracts, including *C. sinensis*, humectants, and abrasive compounds, together with an additional antioxidant component. Also, it proves methods for promoting the oral health of an animal patient.

In 2010, Buzalaf et al. [26] developed a BR patent (PI 1003771–3 A2) filed by the University of São Paulo for dental compositions (gel and/or varnish) containing metalloproteinase inhibitors, such as epigallocatechin-3-galate (EGCC) obtained from *C. sinensis* to be applied in the demineralization of the dental surface, caused by tooth decay or noncarious lesions, as well as preventing the progression of periodontal disease. More recently, in 2020, Juhwan Bio.Cell Company patented a foamable mouthwash solid formulation (WO/2020/054996) [27] which comprises a mixture of green tea and other extracts as an active ingredient. The inventors concluded that the product exhibits an excellent effect on dentin remineralization and dentinal tubule occlusion. Also, the invention has anti-inflammatory, antibacterial, dental caries-preventing, halitosis-eliminating, and scaling effects.

The consumption of *C. sinensis* is a promising agent in maintaining oral health, especially in relation to periodontal disease and caries [1, 7]. Its benefits concerning the prevention of dental caries are due to the leaves being accumulators of fluorine, with antimicrobial action, and the catechins (polyphenols) present have a protective effect on the dental tissue [1, 9, 12, 28]. In addition, the use of *C. sinensis* products for halitosis treatment suggests that this clinical condition can also benefit from these formulations. This fact is due to catechins capable of chemically reacting with sulfur compounds that promote bad breath, through a methylation reaction with orthoquinone, decreasing volatility, and neutralizing the chemical compound, and the antimicrobial activity decreasing the fermentation of sulfur compounds [7, 29]. There is also evidence that these polyphenolic catechins are active in preventing oral cancer and reducing bleeding after tooth extraction [7, 28, 30].

Mouthwash formulations stood out in the present study as a strategy for oral care because of their antimicrobial and anti-inflammatory properties, both in relation to patents and clinical trials found. Radafshar et al. observed the effects of a mouthwash containing 1% green tea tannins on dental biofilm and chronic gingivitis, comparing chlorhexidine and finding similar results [31]. Another clinical trial verified the effect of *C. sinensis* 5% mouthwash on gingivitis induced by biofilm accumulation for five weeks, observing positive effects and no adverse effects [32]. Sarin et al. also evaluated the effectiveness of a mouthwash containing 2% green tea compared to a placebo mouthwash for controlling plaque and gingivitis for four weeks, noting a significant reduction in plaque and gingival index in the group treated with *C. sinensis* mouthwash compared to placebo [33]. Another clinical trial evaluated the effectiveness of a mouthwash with 5% green tea to control pain and trismus associated with acute pericoronitis and compared it to the mouthwash with chlorhexidine, obtaining better results than the same [34]. According to Ardakani et al., *C. sinensis* becomes an alternative to a therapeutic agent carried in mouthwash, given that it has several other therapeutic properties differently from chlorhexidine [35].

In a recent systematic review published, it was found that there is clinical evidence and a favorable safety profile for the use of *C. sinensis* in the form of mouthwash; these formulations are being able to act as antiseptic, antibiofilm, and anti-inflammatory agents. Thus, *C. sinensis* has a favorable phytochemical and pharmacological profile, making it a promising incorporation agent in mouthwashes [3]. These findings justify the highlight of the rinsing formulations in this study.

In view of all these therapeutic benefits of the constituents of *C. sinensis*, despite the small number of patents, it appears that this natural product has great potential for incorporation in dental products for oral care, which can be an attractive cost-effective alternative for consumers.

## 5. Conclusions

The study pointed out a small number of products protected by patents for *Camellia sinensis* for oral care indication, highlighting compositions to be incorporated in formulations, mainly mouthwash. In the methodological parameters of clinical trials carried out with the formulations, the majority pointed out a low risk of bias.

## Figures and Tables

**Table 1:** Study.

	Title	Publication number	Product	Country	Year	Classification IPC	Inventor
1	Oral care composition containing extract of unoxidized *Camellia*	4852/DELNP/2007	Composition	US	2005	A61K	Colgate–Palmolive®
2	Oral compositions containing oxidized *Camellia*	4860/DELNP/2007	Composition	US	2005	A61K/A61Q	Colgate–Palmolive®
3	Oral composition comprising *Camellia* extract of semioxidized tissue from a member of the genus *Camellia* and an enhancing agent	2387/KOLNP/2009	Composition	UK	2006	A01N	Kingston University
4	Composição de higiene oral, método de tratamento ou prevenção de xerostomia e uso de polifenol de chá verde (gtp) (in Portuguese)	BRPI1008380	Composition	US	2008	A01N/A61K	Georgia Health Sciences University Research Institute, Inc.
5	Composição oral, e, métodos para a promoção da saúde oral de um paciente animal, e para a redução da extensão de descoloração em uma pasta de dentes (in Portuguese)	BRPI0519427 (A2)	Toothpaste	US	2004	A61K/A61Q	Colgate–Palmolive®
6	Formulação tópica de uso bucal e seu uso (in Portuguese)	BR102016237505	Composition	BR	2016	A61K/A61P	University of São Paulo
7	Composições odontológicas contendo inibidores de metaloproteinases e seus usos (in Portuguese)	PI 1003771-3	Composition	BR	2010	A61K/A61Q	University of São Paulo
8	Oral care composition	WO2017/199453	Toothpaste	IN	2012	A61K/A61Q	Nippon Zettoc Co., Ltd.
9	Oral care compositions for treating xerostomia	PCT/US2010/024906	Composition	US	2009	A01N/A61K	University of São Paulo
10	Compositions for the treatment and prevention of infections of the oral cavity	PCT/EP2009/002515	Composition	IT	2009	A61K/A61P	Indena SpA Company
11	Noncarious material and anticarious agent containing rare sugar	US 20100166678 A1	Composition	JP	2006	A61K/A61P	Matsutani Chemical Industry Co., Ltd./National University Corporation Kagawa University
12	Antibacterial oral rinse formulation for preventing coronary artery disease	US 20070154414 A1	Mouthwash	US	2005	A61K	Richard Paul Bonfiglio
13	Epigallocatechin-3-gallate crystal compositions	WO2008/153938	Composition	US	2007	C09K/A61P/C07D/A61K	University of South Florida
14	Oral care compositions containing combinations of antibacterial and host-response modulating agents	US 20070053849 A1	Composition	US	2006	A61K/A61P/A61Q	The Procter & Gamble Company
15	Use of a phenol-containing extract from *Camellia sinensis*in oral and dental cleaning agents for improving the visual appearance of the gums	WO/2016/062449	Composition	DE	2016	A61K/A61Q	Henkel Ag & Co., KGaA
16	Oral composition containing saponin extracted from the root of *Camellia sinensis* for effectively preventing or treating periodontal diseases	KR1020130035323	Composition	KR	2013	A61K/A61Q/Y10S	Jeong Kee Kim; Su Kyung Kim; Dae Bang Seo; Seok Sik Moon
17	Composition for enhancing oral hygiene comprising natural extract as active ingredient and use thereof	KR1020160050108	Composition	KR	2016	A61K/A61P/A61Q	Dongguk University Gyeongju Campus Industry-Academy Cooperation Foundation
18	Effervescent mouthwash	US20130149359	Mouthwash	KR	2011	A61K/A61Q	Eric M. Sanders
19	Health care buccal bag for refreshing mouth smell	CN101015516	Bagged tea	CN	2007	A61K/A61Q/A61P/A23F	Zhu Huagang
20	Foamable mouthwash solid formulation and preparation method therefor	WO/2020/054996	Mouthwash	KR	2020	A61K	Eun Sang Lee

## Data Availability

This study used data available in the banks on intellectual property cited on the methods.
